# 

**Published:** 1879-04

**Authors:** 


### THE BUFFALO MEDICAL AND SURGICAL JOURNAL.

VOL. XVIII. APRIL, 1879. NO. 9.

